# Body Iron Stores and Heme-Iron Intake in Relation to Risk of Type 2 Diabetes: A Systematic Review and Meta-Analysis

**DOI:** 10.1371/journal.pone.0041641

**Published:** 2012-07-26

**Authors:** Zhuoxian Zhao, Sheyu Li, Guanjian Liu, Fangfang Yan, Xuelei Ma, Zeyu Huang, Haoming Tian

**Affiliations:** 1 Department of Endocrinology and Metabolism, Sichuan University, West China Hospital, Chengdu, China; 2 Chinese Evidence-Based Medicine/Cochrane Center, Chengdu, China; 3 Department of Oncology, State Key Laboratory of Biotherapy and Cancer Center, West China Medical School, Sichuan University, West China Hospital, Chengdu, China; 4 Department of Orthopedics, Sichuan University, West China Hospital, Chengdu, China; Pennington Biomedical Research Center, United States of America

## Abstract

**Background and Objective:**

Emerging evidence from biological and epidemiological studies has suggested that body iron stores and heme-iron intake may be related to the risk of type 2 diabetes (T2D). We aimed to examine the association of body iron stores and heme-iron intake with T2D risk by conducting a systematic review and meta-analysis of previously published studies.

**Research Design and Methods:**

Systematic review and subsequent meta-analysis were conducted by searching MEDLINE database up to June 22, 2012 to identify studies that analyzed the association of body iron stores or dietary heme-iron intake with T2D risk. The meta-analysis was performed using the effect estimates and 95% confidence intervals (CIs) to calculate the pooled risk estimates, while the heterogeneity among studies was examined using the I^2^ and Q statistic.

**Results:**

The meta-analysis included 16 high-quality studies: 12 studies analyzed ferritin levels (4,366 T2D patients and 41,091 controls) and 4 measured heme-iron intake (9,246 T2D patients and 179,689 controls). The combined relative risk (RR) comparing the highest and lowest category of ferritin levels was 1.66 (95% CI: 1.15–2.39) for prospective studies, 2.29 (95% CI: 1.48–3.54) for cross-sectional studies with heterogeneity (Q = 14.84, p = 0.01, I^2^ = 66.3%; Q = 44.16, p<0.001, I^2^ = 88.7%). The combined RR comparing the highest and lowest category of heme-iron intake was 1.31 (95% CI: 1.21–1.43) with heterogeneity (Q = 1.39, p = 0.71, I^2^ = 0%). No publication bias was found. Additional 15 studies that were of good quality, had significant results, and analyzed the association between body iron stores and T2D risk were qualitatively included in the systematic review.

**Conclusions:**

The meta-analysis and systematic review suggest that increased ferritin levels and heme-iron intake are both associated with higher risk of T2D.

## Introduction

Iron serves as a potent pro-oxidant in human body and participates in the generation of reactive oxygen species (ROS) such as hydroxyl radical [1]. The susceptibility of β-cells to iron-induced oxidative stress and the iron deposition in β-cells usually leads to apoptosis, and consequently, to insulin deficiency [2], [3]. Iron deposition also induces insulin resistance by inhibiting glucose uptake in fat and muscle tissues, and reducing the capacity of liver to extract insulin, which results in an abnormal increase in hepatic glucose production [4]–[6]. The causative role of elevated iron store levels in the onset of insulin resistance is well established by prospective data as well as evidence that blood donations improve insulin sensitivity by decreasing iron stores [7], [8]. Thus, iron deposition and iron-induced oxidative stress contribute to the pathogenesis of type 2 diabetes (T2D) through β-cells apoptosis, hepatic dysfunction, and insulin resistance [9].

Epidemiological studies have suggested a statistically-significant association between ferritin levels and the risk of T2D [10], [11]. Heme-iron intake, the major dietary resource of body iron stores, was also positively associated with T2D risk [12]. Recently, a large number of primary studies regarding ferritin levels and T2D have been published, but a meta-analysis has not yet been conducted to evaluate the available data and the consistency of published primary findings. So far, it is also unclear whether some metabolic factors, such as insulin levels and inflammatory score, serve as confounding factors that significantly change the association of ferritin levels and heme-iron intake with T2D risk [13].

**Figure 1:**
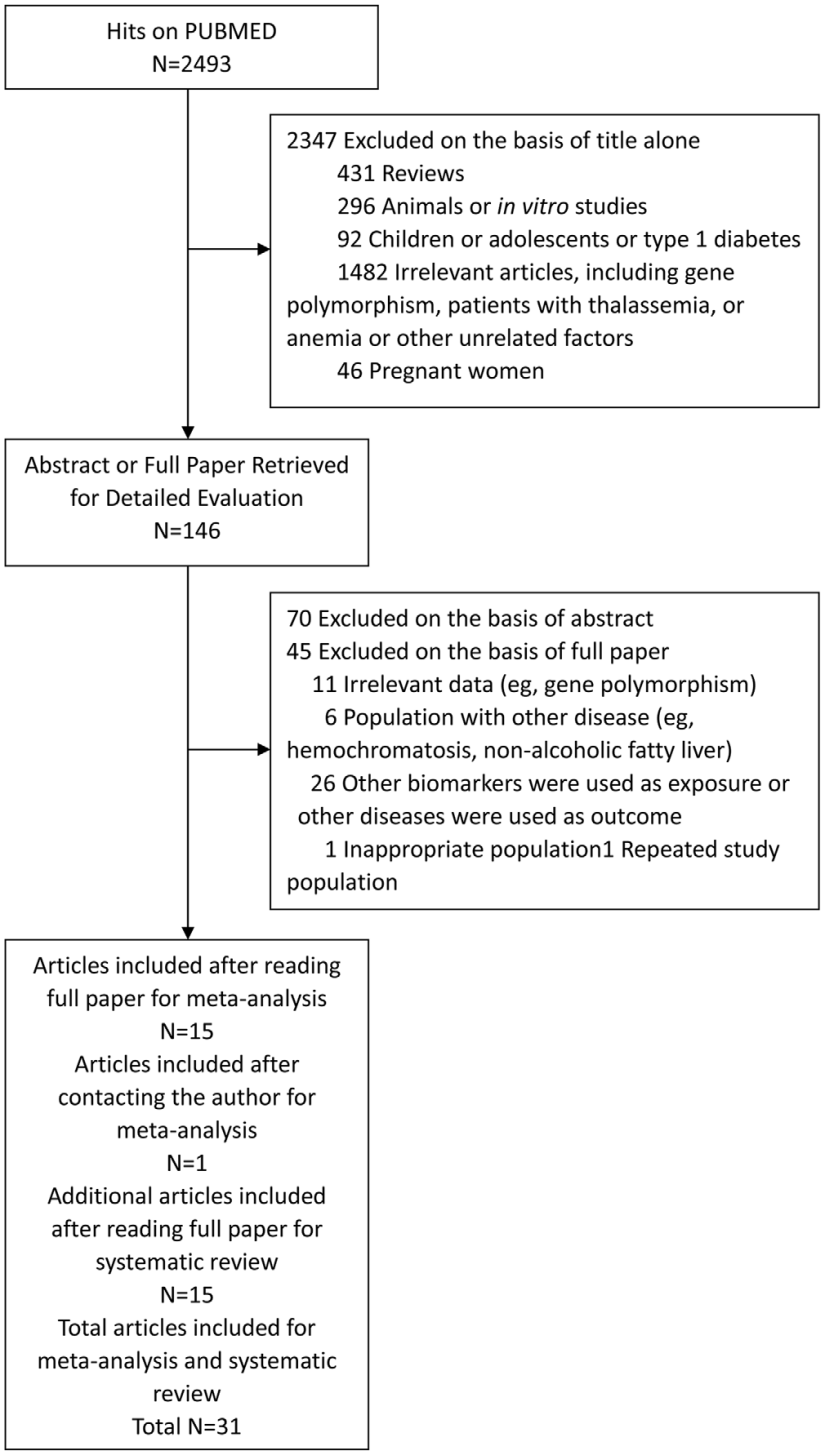
Selection of studies for meta-analysis.

In order to address the need for a cohesive evaluation of existing findings, we performed a systemic review and meta-analysis on the association of body iron stores and heme-iron intake with T2D risk: 1) to summarize the quantitative data respectively from prospective and cross-sectional studies, 2) to qualitatively examine existing studies regarding the association between body iron stores and T2D risk, 3) to examine the association between body iron stores and T2D risk by stratified analysis and meta-regression of parameters, including study design, geographic area, gender, study size, number of patients and controls, metabolic factors, and methods for measuring ferritin levels, and 4) to assess the likelihood of reverse causation and publication bias.

**Table 1:** Characteristics of all identified studies (N = 16) of ferritin levels, iron intake and the risk of type 2 diabetes for the meta-analysis.

First Author Year (ref.)	Country	Study Design	Sex	Age (years)	N/n cases	Ferritin Assay	Follow-up (years)	Diabetes Ascertainment
Jiang et al, 2004 [10]	U.S.	Nested case-control	F	30–55	698/716	TIA	10	Symptoms plus fasting glucose level or random glucose or OGTT or diabetes medication use
Salomaa et al, 2010 [11]	Finland	Cohort	M/F	≥30	179/4798	chemiluminescent microparticleimmunoassay	7.1	Fasting glucose level or diabetes medication use or self-report
Jiang et al, 2004 [12]	U.S.	Cohort	M	40–75	1168/37226	None	12	Symptoms plus fasting glucose level or random glucose or OGTT or diabetes medication use
Jehn et al, 2007 [13]	U.S.	Case-cohort	M/F	45–64	599/690	TIA	7.9	Fasting or nonfasting glucose level or diabetes medication use or self-report
Shi et al, 2006 [19]	China	Cross-sectional	M/F	≥20	79/2770	RIA	None	Fasting glucose level
Kim et al, 2011 [20]	South Korea	Cross-sectional	M/F	20–89	1054/11036	TIA	None	Fasting glucose level or diabetes medication use
Lee et al, 2011 [21]	South Korea	Cross-sectional	M/F	≥20	No data	RIA	None	Fasting glucose level or diabetes medication use
Rajpathak et al, 2009 [22]	U.S.	Nested case-control	M/F	≥25	280/280	TIA	2.8	OGTT or semi-annual fasting glucose
Luan et al, 2008 [23]	China	Cross-sectional	M/F	≥18	147/2850	RIA	None	Fasting glucose level
Ford et al, 1999 [24]	U.S.	Cross-sectional	M/F	≥20	310/9176	RIA	None	Fasting glucose level
Le et al, 2008 [25]	U.S.	Cohort	M/F	20–83	220/5292	RIA	4–5	Fasting glucose level or diabetes medication use or previous diagnosis
Forouhi et al, 2007 [26]	U.K.	Nested case-control	M/F	40–74	360/758	Fluoroimmunoassay	5.1	Self-report from first and second health check and lifestyle questionnaire, diabetes medication use, HbA_1c_
Sun et al, 2008 [27]	China	Cross-sectional	M/F	50–70	440/2725	TIA	None	Fasting glucose level or diabetes medication use or previous diagnosis
Lee et al, 2004 [28]	U.S.	Cohort	F	55–69	1921/26280	None	11	Questions of mailed surveys
Rajpathak et al, 2006 [29]	U.S.	Cohort	F	34–59	4599/80432	None	20	Symptoms plus fasting glucose level or random glucose or OGTT or diabetes medication use

M, male; F, female; OGTT, oral glucose tolerance test; RIA, immunoradiometric assay; TIA, immunoturbidimetric assay.

## Methods

### Search Strategy

Two investigators (Zhuoxian Zhao, Sheyu Li) independently identified articles and reference lists of selected articles in the MEDLINE database through June 2012 using a manual bibliography search. Search terms for MEDLINE were (“ferritin” or “transferrin” or “iron”) and (“diabetes” or “diabetes mellitus”) without any language or article type restriction. Our search included articles that provided at least an abstract, but unpublished reports were not considered. When several studies from the same group analyzed the same set of patients, the articles that had the largest number of T2D cases were selected. The systemic review and meta-analysis was conducted following the Meta-analysis of Observational Studies in Epidemiology (MOOSE) guidelines [14].

**Table 2:** Assessment of quality of all included studies for systematic review and meta-analysis.

1. Assessment of quality of included studies for meta-analysis						
	Selection		Comparability	Exposure/Outcome		Quality of Evidence
Studies	Were characteristics of subjects clearly described?	Were subjects representative of the entire population?	Was the study controlled for confounders adequate?	Was the ascertainment of exposure/outcome clearly described?	Was the follow up long enough?	
Jehn et al, 2007 [13]	Yes	Yes	Yes	Yes	Yes	++++
Jiang et al, 2004 [10]	Yes	Yes	Yes	Yes	Yes	++++
Ford et al, 1999 [24]	Yes	Yes	Yes	Yes	None	+++−
Le et al, 2008 [25]	Yes	Yes	Yes	Yes	Yes	++++
Salomaa et al, 2010 [11]	Yes	Yes	Yes	Yes	Yes	++++
Shi et al, 2006 [19]	Yes	Yes	Yes	Yes	None	+++−
Kim et al, 2011 [20]	Yes	Yes	Yes	Yes	None	+++−
Lee et al, 2011 [21]	Yes	Yes	Yes	Yes	None	+++−
Rajpathak et al, 2009 [22]	Yes	Yes	Yes	Yes	Yes	++++
Luan et al, 2008 [23]	Yes	Yes	Yes	Yes	None	+++−
Forouhi et al, 2007 [26]	Yes	Yes	Yes	Yes	Yes	++++
Sun et al, 2008 [27]	Yes	Yes	Yes	Yes	None	+++−
Jiang et al, 2004 [12]	Yes	Yes	Yes	Yes	Yes	++++
Lee et al, 2004 [28]	Yes	Yes	Yes	Yes	Yes	++++
Rajpathak et al, 2006 [29]	Yes	Yes	Yes	Yes	Yes	++++
Song et al, 2004 [30]	Yes	Yes	Yes	Yes	Yes	++++

2. Assessment of quality of additional included studies for systematic review						
	Selection		Comparability	Exposure		Quality of Evidence
Studies	Were characteristics of subjects clearly described?	Were subjects representative of the entire population?	Was the study controlled for confounders adequate?	Was the ascertainment of the exposure clearly described?	Was follow-uplong enough?	
Kolberg et al, 2009 [31]	Yes	Yes	Yes	Yes	Yes	++++
Jiang et al, 2001 [32]	No	No	Yes	Yes	None	+−−−
Kim et al, 2008 [33]	Yes	Yes	Yes	Yes	None	+++−
Wu et al, 2011 [34]	Yes	Yes	Yes	Yes	None	+++−
Freixenet et al, 2010 [35]	Yes	No	Yes	Yes	None	++−−
Aso et al, 2010 [36]	Yes	No	Yes	Yes	None	++−−
Ashourpour et al, 2010 [37]	No	No	No	Yes	None	+−−−
Kim et al, 2000 [38]	No	No	Yes	Yes	None	+−−−
Hernández et al, 2005 [39]	Yes	No	Yes	Yes	None	++−−
Salonen et al, 1998 [40]	Yes	Yes	Yes	Yes	Yes	++++
Ren et al, 2004 [41]	Yes	Yes	Yes	Yes	None	+++−
Ellervik et al, 2011 [42]	Yes	Yes	Yes	Yes	None	+++−
Mainous ? et al, 2002 [43]	Yes	Yes	Yes	Yes	Yes	++++
Rowe et al, 2012 [44]	Yes	Yes	Yes	Yes	Yes	++++
Hardikar et al, 2012 [45]	Yes	Yes	Yes	Yes	Yes	++++

### Inclusion and Exclusion Criteria

Included studies that were used for meta-analysis met the following criteria: 1) study designs were prospective cohort studies or cross-sectional, 2) the exposure of interest was ferritin or iron intake; 3) the outcome of interest was the prevalence or incidence of T2D, and 4) the effect estimates, assessing the association of ferritin or iron intake levels with T2D risk, and the corresponding 95% confidence interval (CI) were reported. Additional studies, where effect estimates were not provided or could not be calculated based on the provided data, were also included for the systematic review, if they analyzed the association between body iron stores and T2D risk. The systematic review also included studies that provided effect estimates and CIs, even if the number of studies was too small to be included in our meta-analysis.

**Table 3:** Characteristics of additional identified studies (N = 15) of body iron stores and the risk of T2D for the systematic review.

First Author Year (ref.)	Country	Study Design	Sex	N/n cases	Summary of Results
Kolberg et al, 2009 [31]	Denmark	Nested case-control	M/F	160/472	Participants who developed T2D had significantly higher ferritin compared with participants who did not develop T2D (P<0.0001)
Jiang et al, 2011 [32]	China	Case-control	M/F	34/30	T2D had significantly higher ferritin and sTfR levels compared with age-matched controls (P<0.001)
Kim et al, 2008 [33]	U.S.	Cross-sectional	F	244/6015	Women with diabetes had significantly higher ferritin measurements compared with unaffected women (P<0.0001)
Wu et al, 2011 [34]	China	Cross-sectional	M/F	434/2755	Ferritin was independently associated with the prevalence of T2D (P<0.001)
Freixenet et al, 2010[35]	Spain	Case-control	M	51/99	Ferritin, transferrin saturation index and sTfR were not significantly different between men with or without T2D (P = 0.213; 0.624; 0.256 respectively)
Aso et al, 2010 [36]	Japan	Cross-sectional	M/F	104/65	Ferritin was significantly higher in T2D than in controls (P = 0.0055)
Ashourpour et al, 2010 [37]	Iran	Cross-sectional	M/F	54/53	Ferritin, but not iron intake, was significantly associated with T2D (P = 0.048; 0.731 respectively)
Kim et al, 2000 [38]	South Korea	Cross-sectional	M/F	50/25	Log ferritin was higher in T2D than controls with no statistically significant level (P = 0.09)
Hernández et al, 2005[39]	Spain	Case-control	M/F	84/60	Ferritin, but not sTfR, was significantly associated with T2D (P = 0.006; 0.24 respectively)
Salonen et al, 1998 [40]	Finland	Case-control	M	41/82	Men in the lowest quarter of the ratio of transferrin receptors to ferritin were more likely to develop T2D. OR: 2.5 (CI: 1.1–6.0)
Ren et al, 2004 [41]	China	Cross-sectional	M/F	121/85	Ferritin was higher in T2D compared with healthy controls (P<0.05)
Ellervik et al, 2011 [42]	Denmark	Case-control	M/F	5758/34375	Elevated transferrin saturation conferred increased risk of developing T2D. OR: 1.7 (CI: 1.4–2.1)
Mainous III et al, 2002[43]	U.S.	Retrospective cohort	M/F	946/8328	Elevated transferrin saturation level was not significantly associated with the developing of diabetes. OR: 1.03 (CI: 0.44–2.43)
Rowe et al, 2012 [44]	U.S.	Prospective cohort	M/F	127/595	Diabetes converters had significantly higher ferritin levels than non-converters (P = 0.0078)
Hardikar et al, 2012 [45]	India	Prospective cohort	M/F	19/224	Ferritin concentrations were significantly lower in the prediabetic and diabetic compared with the normal group

T2D, type 2 diabetes; M, male; F, female; sTfR, soluble transferrin receptors; OR: odds ratio; CI: confidence interval.

Excluded articles were literature reviews, *in vitro* or animal studies, and studies concerning children, adolescents, pregnant women, type 1 diabetes (T1D), and/or gestational diabetes. We excluded duplicate studies as well as studies regarding gene polymorphism, hemochromatosis, thalassemia, and hepatitis C.

### Data Extraction and the Assessment of Quality

The flow diagram of data extraction is shown in Figure 1. Data were extracted independently by two researchers (Zhuoxian Zhao, Sheyu Li) using a standardized data extraction form. Discrepancies were resolved by consulting a third investigator (Guanjian Liu), who is an expert in evidence-based medicine. Corresponding or first authors of studies, which had potentially collected relevant data, but didn’t provide odds ratio (OR) or relative risk (RR) or hazard risk (HR) values for T2D risk, were contacted through e-mail to inquire about the raw data.

The following data was extracted from the included studies: first author, publication year, country, study design, gender and age of patients, number of T2D patients and controls, criteria for T2D diagnosis, effect estimate and corresponding 95% Cl, duration of follow-up, and adjustments for confounding factors. For the different categories of exposure, such as the tertiles, quartiles or quintiles, we extracted the effect estimates and corresponding 95% CI comparing the highest versus lowest category of ferritin levels or heme-iron intake for T2D risk.

**Table 4:** Effect Estimates of type 2 diabetes according to ferritin levels and dietary iron intake in all 16 studies for the meta-analysis.

Source	Model	Comparison and Effect Estimates (95% CI)	Adjustment for Covariates
Jiang et al, 2004 [10]	Model 1^b^	RR: Ferritin: 2.61 (1.78, 3.85)	Age, race, fasting status, BMI
	Model 2	RR: Ferritin: 2.61 (1.68, 4.07), Premenopausal women: 3.08 (1.11, 8.53), Postmenopausal women: 2.17 (1.20, 3.93)	Model 1 covariates + diabetes family history, physical activity, smoking, drinking, menopausal status, glycemic load, intake of total energy, cereal fiber, magnesium, *trans*-fat, ratio of polyunsaturated fat to saturated fat
Salomaa et al, 2010 [11]	Model 1^b^	HR: Ferritin: 1.44 (0.93, 2.24)	Sex, non-HDL-C, HDL-C, triglyceride, BMI, systolic blood pressure, smoking, blood glucose, history of cardiovascular disease event and use of antihypertensive medication
Jiang et al, 2004 [12]	Model 1^b^	RR: Total iron intake: 0.87 (0.71, 1.05), Heme iron intake: 1.47 (1.21, 1.79)	Age, BMI
	Model 2	RR: Total iron intake: 1.16 (0.92, 1.47), Heme iron intake: 1.28 (1.02, 1.61)	BMI, diabetes family history, physical activity, smoking, drinking, intakes of total energy, *trans* fat, cereal fiber, magnesium, whole grains, fruit, vegetables, ratio of polyunsaturated fat intake to saturated fat intake, glycemic load, multivitamin use
Jehn et al, 2007 [13]	Model 1^b^	HR: Plasma ferritin: 1.51 (0.98, 2.31)	Age, center, ethnicity, smoking, alcohol, BMI
	Model 2	HR: Plasma ferritin: 0.79 (0.48, 1.32)	Model 1 covariates + HDL-C, waist circumference, hypertension, fasting glucose level, fasting triglyceride, fasting insulin level, inflammation score
	Model 3	HR: Plasma ferritin: Men: 0.89 (0.66, 1.20), Postmenopausal women: 0.99 (0.71, 1.36), Premenopausal women: 0.85 (0.62, 1.16)	Gender, menopausal status, age, race, smoking, drinking, BMI, hypertension, HDL-C, fasting triglyceride, fasting glucose, fasting insulin, inflammation score
Shi et al, 2006 [19]	Model 1^b^	OR: Serum ferritin^a^, Men: 1.08 (0.50, 2.33); Women: 4.07 (1.86, 8.90), OR: Total iron intake, Men: 1.54 (0.44, 5.37); Women: 5.53 (1.47, 20.44)	Age, BMI, central obesity, smoking, drinking, diabetes family history, high blood pressure, urban income, job, education, intake of iron, energy, protein, fat
	Model 2	OR: Serum ferritin^a^: 1.98 (1.19, 3.29), OR: Total iron intake: 3.73 (1.50, 9.26)	Model 1 covariates + high hemoglobin, high ferritin, sex
Kim et al, 2011 [20]	Model 1^b^	OR: Serum ferritin: Men: 1.71 (1.38, 2.12), Women: 1.50 (1.05, 2.13)	Age
	Model 2	OR: Serum ferritin: Men: 1.27 (1.01, 1.60), Women: 1.12 (0.76, 1.63)	Model 1 covariates + BMI, waist circumference, systolic and diastolic blood pressure, triglyceride, HDL-C, cholesterol, hsCRP, smoking, alcohol use, menopause use, AST, ALT, GGT
Lee et al, 2011 [21]	Model 1^b^	OR: Serum ferritin: Men: 1.80 (1.09, 2.97), Premenopausal women: 3.57 (1.38, 9.21), Postmenopausal women: 1.54 (0.90, 2.65)	Age, education level, smoking, alcohol intake, BMI, AST, ALT
Rajpathak et al, 2009 [22]	Model 1^b^	OR: Ferritin: 1.02 (0.60, 1.74)	Age, sex, race, BMI
	Model 2	OR: Ferritin: 1.61 (0.85, 3.02)	Model 1 covariates + diabetes family history, physical activity, glycated hemoglobin, sTRF
Luan et al, 2008 [23]	Model 1^b^	OR: Ferritin: 4.34 (2.31, 8.14), OR: Heme iron intake: 2.62 (1.56, 4.40)	Age, sex
	Model 2	OR: Ferritin: 2.96(1.53, 5.72), OR: Heme iron intake: 2.30 (1.26, 4.19)	Model 1 covariates + smoking, drinking, sedentary time, diabetes family history, central obesity, high blood pressure, abnormal blood lipid, intake of calories, fiber, high percentage of energy from fat
Ford et al, 1999 [24]	Model 1^b^	OR: Ferritin: Men: 4.94 (3.05, 8.01), Women: 3.61 (2.01, 6.48)	Age, sex, ethnicity, education, BMI, drinking, alanine aminotransferase, CRP, examination session attended
Le et al, 2008 [25]	Model 1^b^	HR: Ferritin: Men: 1.79 (1.13, 2.82), Women: 0.87 (0.37, 2.03)	BMI, age, race
Forouhi et al, 2007 [26]	Model 1^b^	OR: Ferritin: 7.4 (3.5, 15.4)	Age, BMI, diabetes family history, physical activity, smoking, dietary factors
	Model 2	OR: Ferritin: 3.2 (1.3, 7.6)	Model 1 covariates + CRP, fibrinogen, IL-6, liver function tests, adiponectin
Sun et al, 2008 [27]	Model 1^b^	OR: Ferritin: 3.06 (2.20, 4.27)	Age, sex, region, smoking, drinking, physical activity, education, diabetes family history, dietary factors, use of iron supplements, BMI
	Model 2	OR: Ferritin: 2.76 (1.96, 3.90)	Model 1 covariates + inflammatory factors, adipokines
Lee et al, 2004 [28]	Model 1^b^	RR: non-heme iron intake: 0.80 (0.64–1.01), heme iron intake: 1.28 (1.04–1.58), Supplemental iron: 1.16 (0.92–1.46)	Age, total energy intake, WHR, BMI, physical activity, smoking, drinking, education, marital status, residential area, hormone replacement therapy, animal fat, vegetable fat, cereal fiber, dietary magnesium, dietary non-heme iron, dietary heme iron, supplemental iron
Rajpathak et al, 2006 [29]	Model 1^b^	RR: Total iron intake: 1.02 (0.90–1.15), Heme iron intake: 1.28 (1.14–1.45)	Age, BMI, diabetes family history, smoking, drinking, physical activity, hormone replacement therapy, multivitamin use, calories, cereal fiber, magnesium, ratio of polyunsaturated fat intake to saturated fat intake, glycemic load, caffeine, trans fat
Song et al, 2004 [30]	Model 1	RR: Total iron intake: 1.13 (0.93–1.37), Heme iron intake: 1.46 (1.20–1.78)	Age, BMI, total energy intake, smoking, exercise, drinking, diabetes family history, dietary intakes of fiber intake, glycemic load, magnesium, total fat

a Quartile 4 vs 1–3 serum ferritin.

b Effect estimates used in the main analysis. CI, confidence interval; HR, hazard ratio; OR, odds ratio; RR, relative risk; BMI, body mass index; HDL-C, high-density lipoprotein cholesterol; AST, aspartate aminotransferase; ALT, alanine transaminase; GGT, gamma-glutamyl transpeptidase; hsCRP, high sensitivity C-reactive protein; sTRF, serum transferrin receptor-ferritin; IL-6, Interleukin-6; WHR, waist-hip ratio.

**Figure 2:**
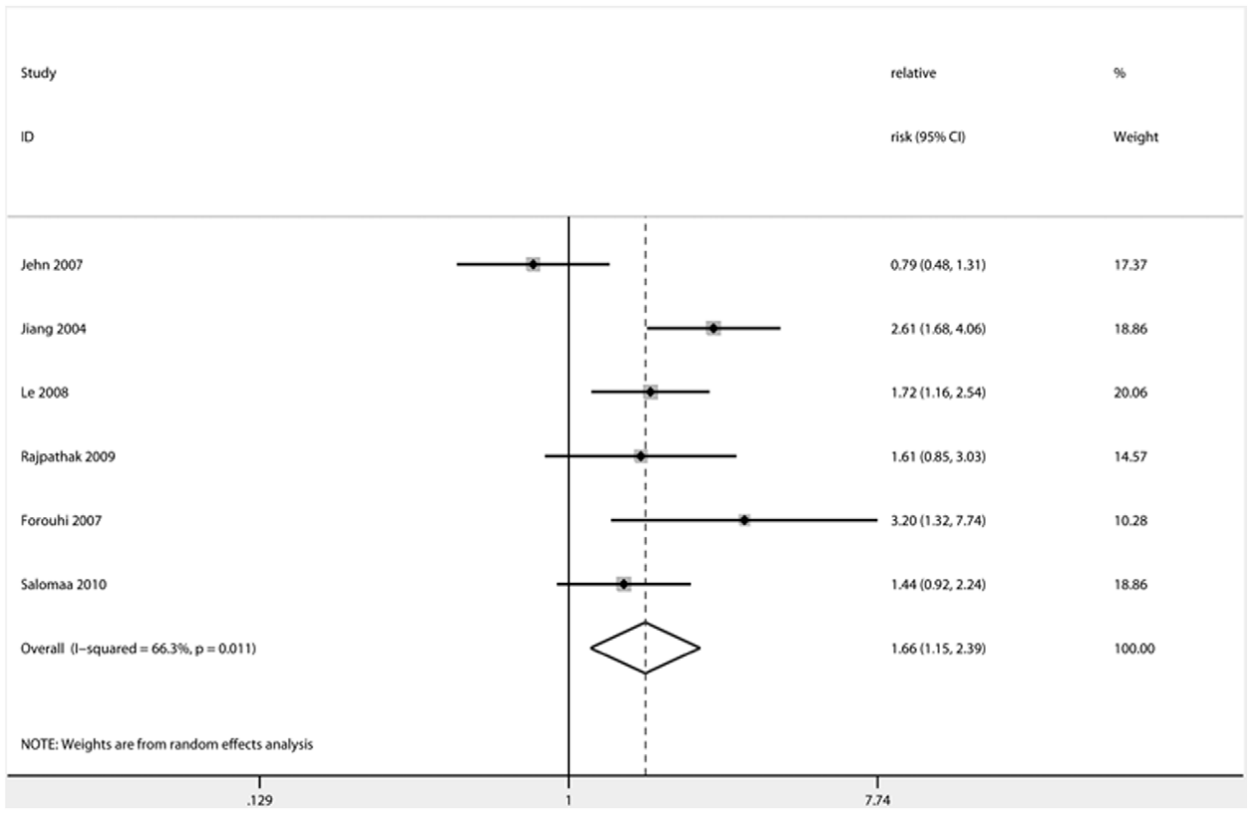
Forest plot showing the effect estimates of each prospective study and the pooled relative risk comparing the highest with the lowest category of ferritin levels. *dotted line represented the combined effect estimate of meta-analysis. Size of square and rhomboids represented weight.

**Figure 3:**
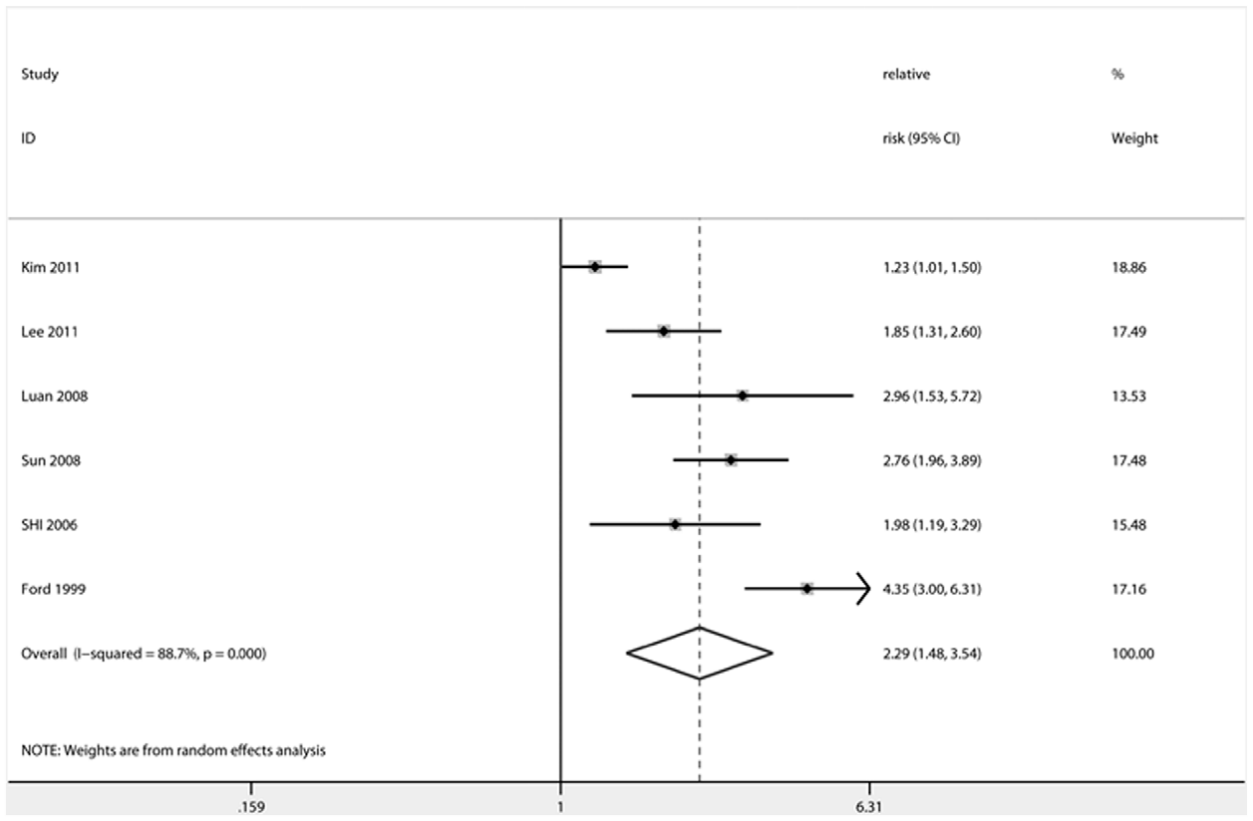
Forest plot showing the effect estimates of each cross-sectional study and the pooled relative risk comparing the highest with the lowest category of ferritin levels. *dotted line represented the combined effect estimate of meta-analysis. Size of square and rhomboids represented weight.

**Figure 4:**
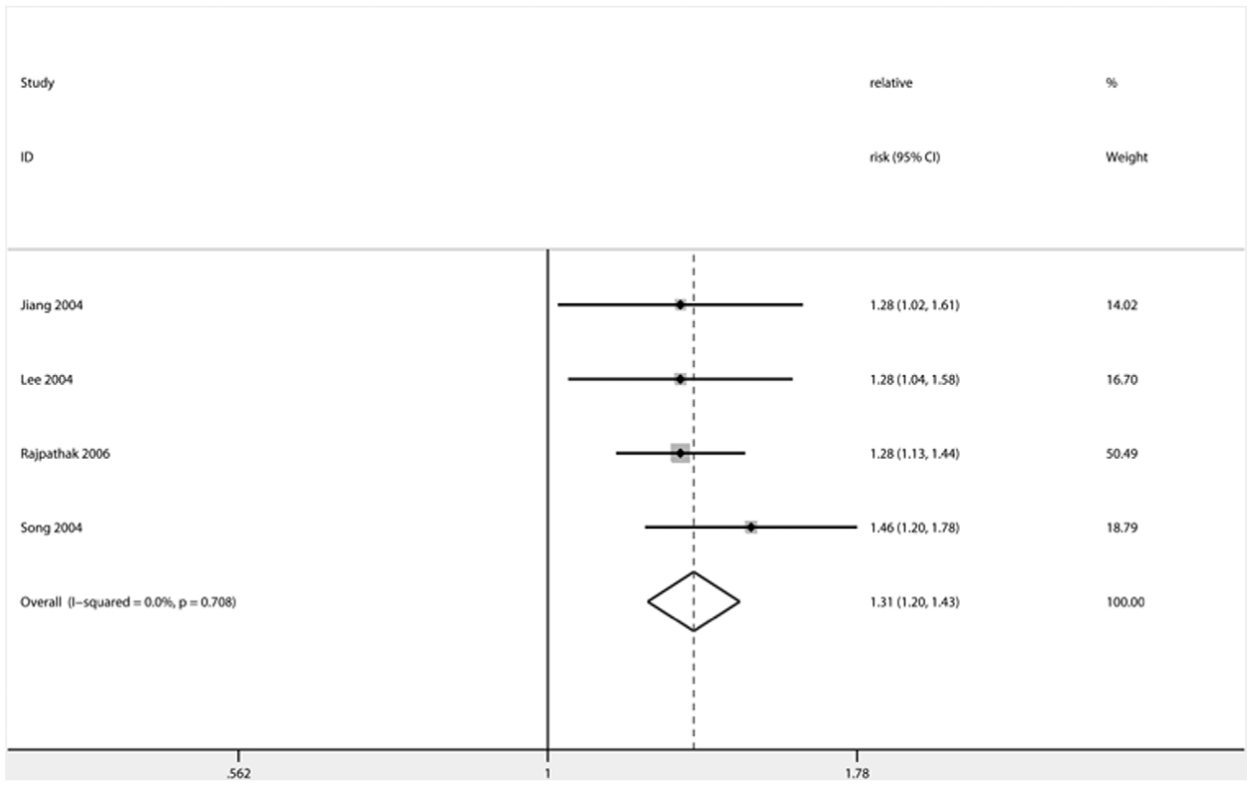
Forest plot showing the effect estimates of each cohort study and the pooled relative risk comparing the highest with the lowest category of heme-iron intake levels. *dotted line represented the combined effect estimate of meta-analysis. Size of square and rhomboids represented weight.

The quality of all included studies was assessed based on the Newcastle-Ottawa scale by determining the selection of participants, adjustments of confounders, description of outcomes, and the duration of follow-up [15]. The approach of the grades of recommendation assessment, development and evaluation (GRADE) approach was used to evaluate the evidence of included studies [16]. The following symbols were chosen to represent the four levels of the quality of evidence: ++++ (high), +++− (moderate), ++−− (low), and +−−− (very low) quality.

**Table 5:** Stratified meta-analysis of ferritin levels and the risk of type 2 diabetes.

Subgroup	Number of studies	Relative risk (95% CI)	Q statistic	P for heterogeneity	I^2^
Prospective Studies					
Study Design					
Nested case-control	3	2.35 (1.68–3.28)	2.05	0.358	2.6%
Cohort	2	1.59 (1.19–2.13)	0.34	0.559	0.0%
Geographic area					
Western	6	1.66 (1.15–2.39)	14.84	0.011	66.3%
Sex					
Men	2	1.23 (0.62–2.45)	6.28	0.012	84.1%
Women	3	1.25 (0.64–2.46)	11.31	0.003	82.3%
** **Study size, cases					
<300	3	1.59 (1.22–2.08)	0.34	0.842	0.0%
≥300	3	1.82 (0.74–4.45)	14.4	0.001	86.1%
** **Adjusted for metabolic factors					
Yes	4	1.49 (0.90–2.46)	12.26	0.007	75.5%
No	2	1.90 (1.33–2.73)	1.59	0.21	37.2%
** **Ferritin assay					
TIA	3	1.50 (0.71–3.15)	12.16	0.002	83.6%
Cross-sectional studies					
Geographic area					
Asia	5	1.98 (1.36–2.88)	21.16	<0.001	81.1%
** **Sex					
Men	4	1.89 (0.97–3.69)	26.03	<0.001	88.5%
Women	4	2.24 (1.23–4.09)	15.56	0.001	80.7%
Study size, cases					
<300	2	2.30 (1.54–3.44)	0.90	0.344	0.0%
≥300	3	2.42 (1.10–5.35)	42.06	<0.001	95.2%
Adjusted for metabolic factors					
Yes	3	1.80 (1.07–3.04)	8.43	0.015	76.3%
No	3	2.80 (1.74–4.51)	11.02	0.004	81.8%
Ferritin assay					
TIA	2	1.82 (0.82–4.02)	16.04	<0.001	93.8%
RIA	4	2.62 (1.66–4.13)	12.43	0.006	75.9%

CI, confidence interval; RIA, immunoradiometric assay; TIA, immunoturbidimetric assay.

### Statistical Analyses

STATA v11.0 (Stata-Corp, College Station, TX, USA) was used for all statistical analyses. All tests were two-tailed and p<0.05 was considered statistically significant. All ORs, RRs, and HRs were considered to be estimates of RR used in the meta-analysis. Homogeneity was tested using the Q test and I^2^ statistic, which represented the variation of effect sizes due to genuine differences across studies rather than chance [17]. A random-effects model described by DerSimonian and Laird was performed to calculate the summarized estimates and corresponding 95% CIs, when significant between-study heterogeneity existed. Alternatively, a fixed effects model was used, when there was no significant heterogeneity across studies. To explore the sources of between-study heterogeneity, subgroup analyses were performed based on study design, geographic area, patient gender, study size, and metabolic factor adjustments. Prospective studies and cross-sectional studies were separately combined in the meta-analysis. Prospective studies, including nested case-control and cohort studies, were separately combined in the subgroup analyses. Sensitivity analyses were also performed to identify the influence of individual studies on the pooled RRs. Both single and multiple models of meta-regression were used to analyze the effect of each covariate on the summarized results and the potential sources of heterogeneity.

**Table 6:** β coefficients and corresponding p values analyzed by meta-regression models.

	Single covariate			Multiple covariates	
Covariate	Number of studies	β coefficient	P value	β coefficient	P value
Prospective Studies	6				
Study Design					
(Nested case-control, Cohort,Case-cohort)	6	−0.514	0.026	−0.627	0.164
Sample Size, cases					
(<300 vs. ≥300)	6	−0.108	0.813	0.069	0.98
Number of cases	6	0.00003	0.976	0.0002	0.98
Number of controls	6	−0.00002	0.877	−0.00007	0.84
Adjusted for metabolic factors(Yes vs. No)	6	−0.367	0.450	−0.556	0.71
Cross-sectional studies	6				
Country	6	−0.798	0.125	NA	NA
(western vs. Asian)					
Sample Size, cases	5*	−0.006	0.991	−0.751	0.31
(<300 vs. ≥300)					
Number of cases	5*	−0.0009	0.195	−0.002	0.20
Number of controls	5*	−0.00003	0.656	0.00003	0.65
Adjusted for metabolic factors(Yes vs. No)	6	−0.446	0.281	NA	NA
Ferritin Assay (TIA vs. RIA)	6	−0.374	0.389	NA	NA

* One study did not provide the data for the number of patients and controls [18]. Each meta-regression model included each covariate as the explanatory variable, and the log relative risk (RR) as the outcome variable. β coefficient represents the change in log RR per unit increase in the relevant variable. NA means the observations were insufficient for calculated.

Publication bias was assessed using the visual inspection of funnel plots, Begg’s rank correlation, and the Egger weighted regression method (p<0.05 was considered statistically-significant for publication bias). A Galbraith plot was also constructed for visual observation of the outlier studies [18].

## Results

### Searching Process

The flow diagram of the literature search strategy is shown in Figure 1. 2,493 articles and abstracts were identified by initial searches, of which 2,347 articles were excluded by manual screening of the titles. An additional 70 papers were excluded after reading the abstract, leaving 76 articles for full publication review. Of these, 15 articles were included after reading the full paper [10], [12], [13], [19]–[30]. Another 16 articles compared ferritin levels or iron intake between T2D patients and controls, but failed to report ferritin levels or iron intake as the exposure to allow the comparison between the highest and lowest category of ferritin levels or iron intake for T2D risk. Therefore, we contacted the authors of these 16 studies and were able to obtain the original data from one author to include in our meta-analysis [11]. Table 1 shows the detailed characteristics of the resulting 16 studies that were included in our meta-analysis.

The systematic review was based on 31 studies, containing additional 15 articles that analyzed the association of ferritin, sTfR (soluble transferrin receptor), transferrin, iron intake, the ratio of transferrin receptors to ferritin or transferrin saturation with T2D risk [31]–[45]. Although the 15 articles that were included for qualitative analysis did not provide the data for effect estimates and corresponding 95% CIs, they analyzed the association between body iron stores and T2D risk, making them eligible to be included in our systematic review. Table S1 showed Preferred Reporting Items for Systematic Reviews and Meta-analyses (PRISMA) checklist.

**Figure 5:**
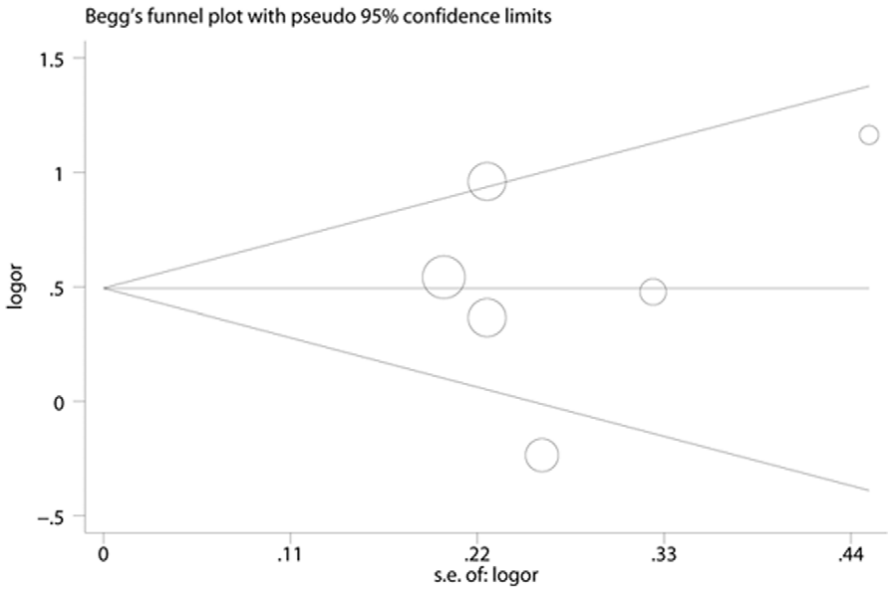
Begg’s Funnel Plots for visual assessment of the presence of publication bias for 6 prospective studies of ferritin in the meta-analysis. Begg’s bias (P = 0.851).

**Figure 6:**
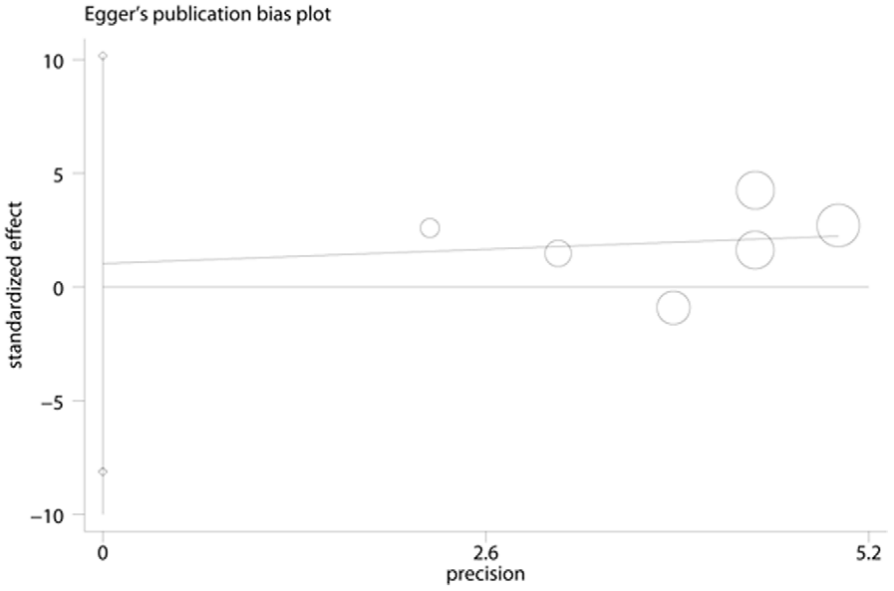
Egger’s Funnel Plots for visual assessment of the presence of publication bias for 6 prospective studies of ferritin in the meta-analysis. Egger’s bias (P = 0.772).

**Figure 7:**
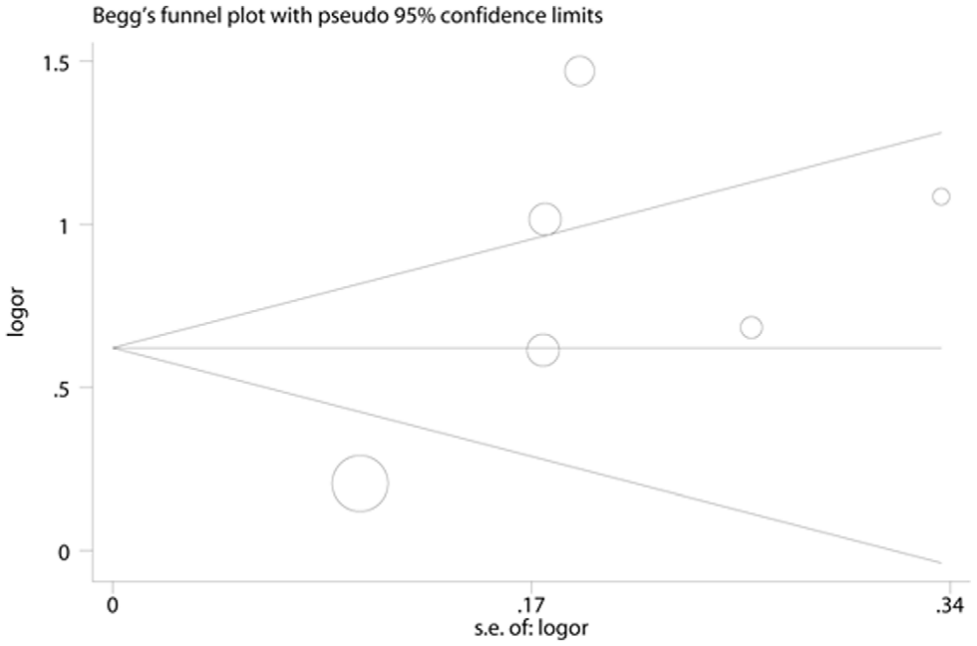
Begg’s Funnel Plot for visual assessment of the presence of publication bias for 6 cross-sectional studies of ferritin in the meta-analysis. Begg’s bias (P = 0.188).

**Figure 8:**
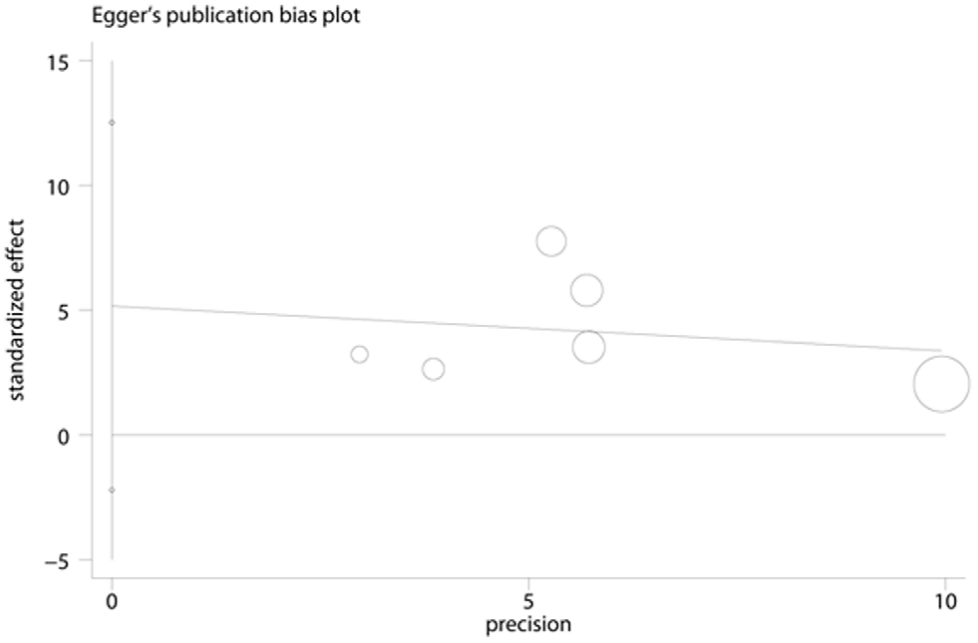
Egger’s Funnel Plot for visual assessment of the presence of publication bias for 6 cross-sectional studies of ferritin in the meta-analysis. Egger’s bias (P = 0.124).

**Figure 9:**
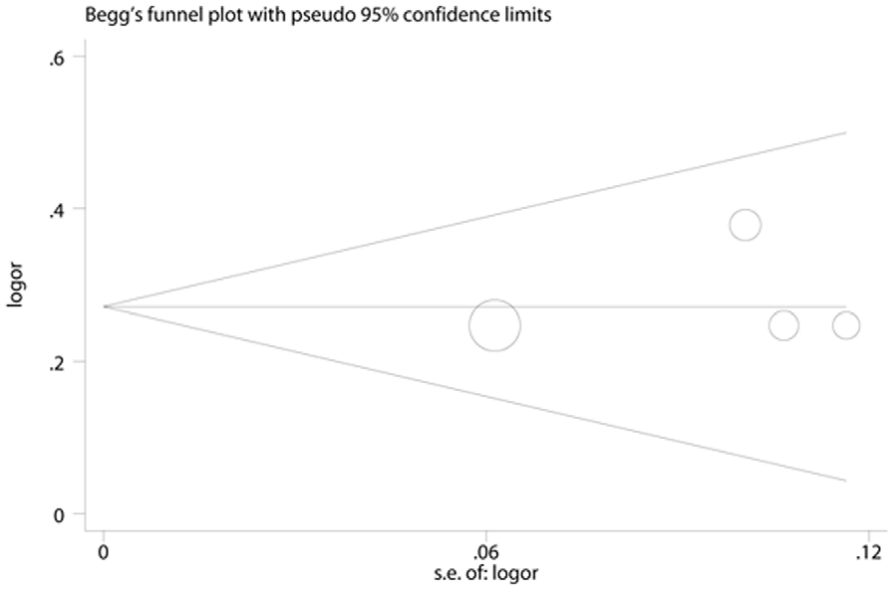
Begg’s and Funnel Plot for visual assessment of the presence of publication bias for 4 cohort studies of heme-iron intake in the meta-analysis. Begg’s bias (P = 0.497).

**Figure 10:**
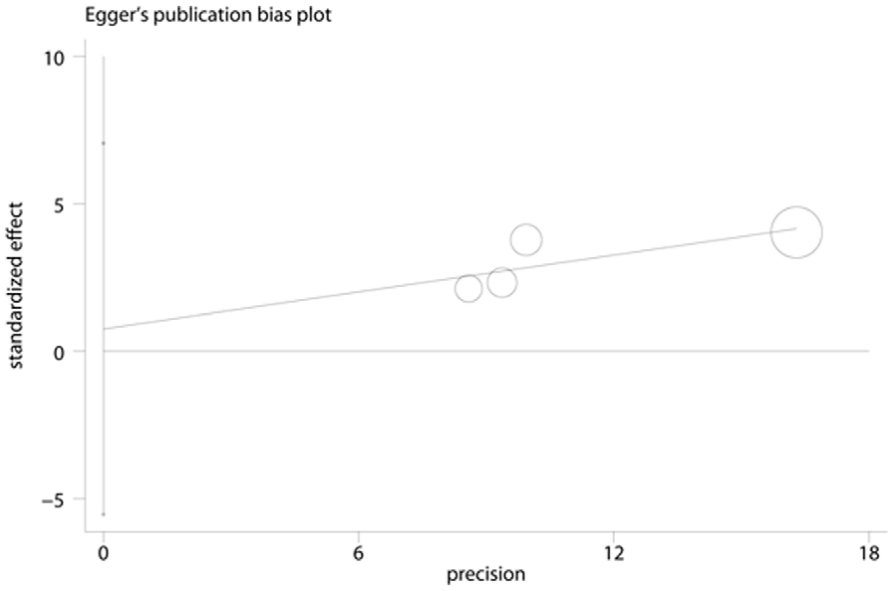
Egger’s Funnel Plot for visual assessment of the presence of publication bias for 4 cohort studies of heme-iron intake in the meta-analysis. Egger’s bias (P = 0.658).

**Figure 11:**
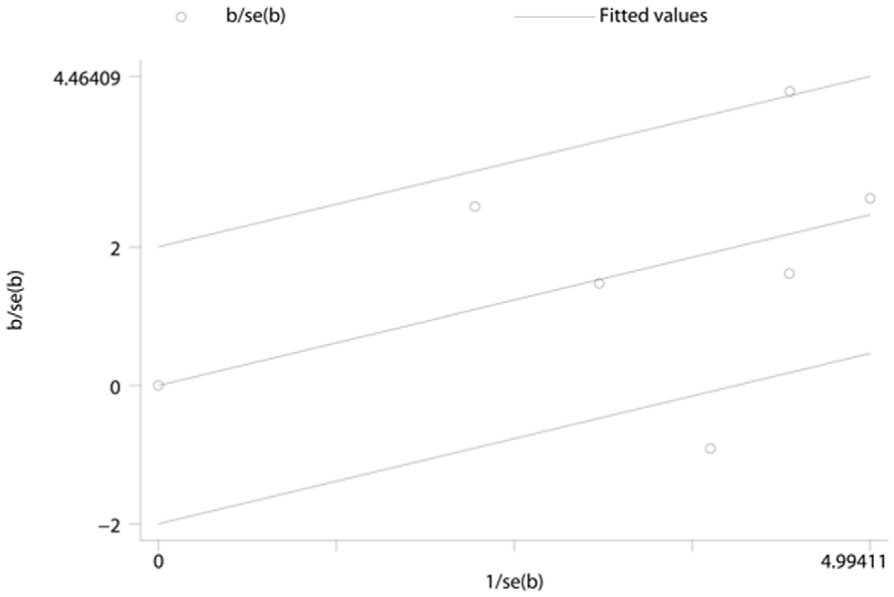
Galbraith plot of the ferritin levels for the association with type 2 diabetes for prospective studies. The regression runs through the origin interval (central solid line). Between the two outer parallel lines is the 95% confidence interval.

**Figure 12:**
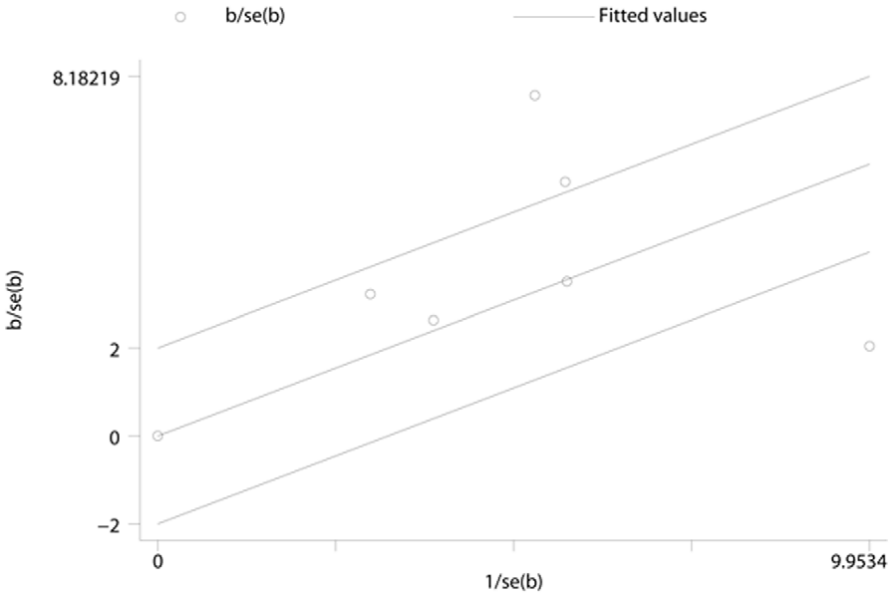
Galbraith plot of the ferritin levels for the association with type 2 diabetes for cross-sectional studies. The regression runs through the origin interval (central solid line). Between the two outer parallel lines is the 95% confidence interval.

### Quality Assessment

The results of the quality assessment are shown in Table 2. Every study included in the meta-analysis had a moderate-to-high quality with the appropriated selection of subjects, adequate adjustment for potential confounders, and clearly described exposure or outcome. Among the additional 15 studies that were included only for the qualitative analysis, 9 studies had moderate-to-high quality, 3 showed low quality, and the remaining 3 had very low quality.

### Study Characteristics

For the meta-analysis, 12 studies assessing ferritin levels (4,366 T2D patients and 41,091 controls) and 4 studies assessing heme-iron intake (9,246 T2D patients and 179,689 controls) met the inclusion criteria. These 16 studies consisted of 6 cohort, 1 case-cohort, 3 nested case-control, and 6 cross-sectional studies. Among the 12 studies assessing the association between ferritin levels and T2D risk, 7 studies were conducted in western countries (5 in North America, 2 in Europe) and 5 in Asia. Furthermore, 11 of these 12 studies consisted of men and women, while 1 study consisted entirely of women [10]. In the 12 ferritin studies, all effect estimates and corresponding CIs were adjusted for confounders. In addition, the results of 4 studies were adjusted for metabolic factors including high-density lipoprotein cholesterol (HDL-C), triglyceride, C-reactive protein (CRP), adiponectin, adipokines, liver enzymes, inflammation score, insulin levels and dietary factors. Among the 6 studies assessing the association between iron intake and T2D risk, 5 studies provided the data assessing the association between heme-iron intake and T2D. Of these iron intake studies, 5 analyzed the association between total iron intake (non-heme, heme and supplemental iron intake) and T2D risk, 1 consisted entirely of men [12], 3 entirely of women [28]–[30]. Furthermore, 4 of these were cohort studies that were quantitatively analyzed in the meta-analysis [12], [28]–[30].

The 15 studies that were included for the qualitative analysis of the systematic review consisted of one nested case-control study, one retrospective cohort, 5 case-control, 2 prospective cohort, and 6 cross-sectional studies (Table 3). Of these, 12 studies measured the body iron store indexes without providing the effect estimates, including ferritin (n = 12), sTfR (n = 3), transferrin saturation index (n = 1), and iron intake (n = 1). Also, one study provided OR and CI, comparing the ratio of transferrin receptors to ferritin with T2D risk, and 2 studies provided ORs and CIs comparing transferrin saturation with T2D risk. Of these 15 studies, 8 were conducted in western countries (3 in North America, 5 in Europe), and 7 in Asia. Also, 12 studies consisted of men and women, one consisted entirely of women [33], and 2 entirely of men [35], [40].

### Main Analysis and Summary of Results

The ORs, HRs or RRs that were maximally adjusted for confounders, including HDL-C, fasting and 2-h glucose, triglyceride, CRP, adiponectin, adipokines, liver enzymes, insulin level and inflammation score, were used for the meta-analysis. Result of the association of ferritin levels and dietary iron intake with the risk of T2D in the meta-analysis studies is presented in Table 4. The effect estimates, the corresponding CIs, and the pooled RRs are shown in Figure 2, 3 and 4 for prospective, cross-sectional studies of ferritin and cohort studies of heme-iron intake, respectively. The combined RR comparing the highest and lowest category of ferritin levels for T2D risk was 1.66 (95% CI: 1.15–2.39) in prospective studies and 2.29 (95% CI: 1.48–3.54) in cross-sectional studies. The combined RR comparing the highest and lowest category of heme-iron intake for T2D risk was 1.31 (95% CI: 1.21–1.43).

Results of the studies that were included for the qualitative analysis of the systematic review were summarized as follows: 1) ferritin levels were significantly higher in T2D patients than in controls in 8 studies, while they were not significantly different between T2D patients and the controls in 2 studies [35], [38], 2) one study suggested significantly higher levels of sTfR in T2D patients compared to control individuals [32], which was not confirmed by 2 other relevant studies [35], [39], 3) transferrin saturation index and iron intake were also not significantly different between patients and control individuals in 2 studies [35], [37], 4) one study provided OR value confirming a positive association between the ratio of transferrin receptors to ferritin and T2D risk [40], and 5) inconsistent results were reported in 2 studies that used ORs and corresponding CIs to analyze the association between transferrin saturation and T2D risk [42], [43].

### Heterogeneity

Moderate heterogeneity among prospective cohort studies (Q = 14.84, P = 0.01, I^2^ = 66.3%), and high heterogeneity among cross-sectional studies (Q = 44.16, P<0.001, I^2^ = 88.7%) were detected for studies of ferritin levels. Among the studies concerning heme-iron intake levels, on the other hand, no heterogeneity was observed (Q = 1.39, P = 0.71, I^2^ = 0%). A sensitivity analysis was conducted by omitting 1 study at a time and calculating the pooled RRs for the remaining studies to identify any potential source of heterogeneity. Sensitivity analysis suggested that no single study dramatically influenced the pooled RRs, which ranged from 1.45 (1.15–1.82) to 1.88 (1.51–2.35) comparing the highest versus the lowest category of ferritin levels for T2D risk among prospective studies, 1.98 (1.36–2.88) to 2.65 (1.90–3.70) among cross-sectional studies, and 1.28 (1.16–1.41) to 1.35 (1.19–1.52) for heme-iron intake among prospective cohort studies.

We also performed a stratified analysis to identify potential sources of heterogeneity. For prospective studies, the positive association between ferritin levels and T2D risk was consistently observed when studies were stratified by study design and patient gender, but was not observed when studies were stratified by study size and adjustments of metabolic factors. On the other hand, for cross-sectional studies, the positive association was consistently observed when studies were stratified by study size and metabolic factors, but not observed when stratified by patient gender and ferritin measuring methods (Table 5).

Both single and multiple covariate models of meta-regression were performed using covariates of sample size, number of patients, number of controls and adjustments of metabolic factors for prospective studies, and covariates of country, sample size, number of patients, number of controls, adjustments of metabolic factors, and methods of ferritin measurement for cross-sectional studies. Results of meta-regression models including the number of included studies, β coefficients and corresponding p values are shown in Table 6.

### Publication Bias

No evidence of publication bias was observed assessing the association between ferritin levels and T2D, as indicated by Begg’s rank correlation and Egger linear regression tests for prospective studies (Figure 5, Begg: P = 0.851; Figure 6, Egger: P = 0.772) and for cross-sectional studies (Figure 7; Begg: P = 0.188; Figure 8, Egger: P = 0.124). Publication bias also did not exist in the association between heme-iron intake and T2D (Figure 9; Begg: P = 0.497; Figure 10, Egger: P = 0.658). Furthermore, Galbraith plot was also used to detect outliers for the studies assessing the association between ferritin and T2D risk. Among the prospective studies (Figure 11), 4 were inside the 95% bounds, 2 were outliers [10], [13] in the Galbraith plot, while among the cross-sectional studies (Figure 12), 3 were within the 95% bounds, and the other 3 were the outliers [20], [24], [27]. However, after removing all the outliers, the pooled RRs were just slightly increased from 1.66 (95% CI: 1.15–2.39) to 1.69 (95% CI: 1.31–2.18) for the prospective studies, and 2.29 (95% CI: 1.48–3.54) to 2.03 (95% CI: 1.56–2.63) for the cross-sectional studies, suggesting that the study results were homogenous in nature.

## Discussion

### Summary of Findings

Our study provided quantitative pooled estimates of the association of T2D risk with ferritin and dietary heme-iron intake for the first time. Our meta-analysis indicated a significant association between ferritin levels and the risk of T2D, which were performed separately among 6 prospective studies and 6 cross-sectional studies (4,366 T2D cases and 41,091 controls). A significant association between dietary heme-iron intake and the risk of T2D was also observed from 4 prospective cohort studies (9,246 T2D patients and 179,689 controls). The initial results of the meta-analysis were consistent with the results of sensitivity analyses. The qualitative analysis of additional studies suggested a statistically significant association between ferritin and T2D risk in 8 out of 10 studies.

### Systematic Review of Current Epidemiological Findings

#### Cross-sectional and case-control findings

We found that the cross-sectional studies (1 in U.S., 2 in South Korea, and 3 in China) indicated a statistically significant association between ferritin and T2D risk after adjustments for multiple confounders, with the effect estimates ranging from 1.23 (1.01–1.50) to 4.35 (3.00–6.31) [19]–[21], [23], [24], [27]. Female T2D patients [2.24 (1.23–4.09)] exhibited a higher combined effect estimate than male patients [1.89 (0.97–3.69)], suggesting that hormonal differences may play a role in the association between ferritin levels and T2D risk. When metabolic factors such as hypertension, triglyceride levels, and waist circumference were adjusted, the combined effect estimate was reduced from [2.80 (1.74–4.51)] to [1.80 (1.07–3.04)]. Different methods of ferritin measurement, namely the immunoradiometric assay (RIA) and immunoturbidimetric assay (TIA), also had a significant impact on the association between ferritin levels and T2D risk (RIA: 2.62, 95% CI: 1.66–4.13; TIA: 1.82, 95% CI: 0.82–4.02). The qualitative analysis of six cross-sectional studies from the U.S., China, Japan, Iran, and South Korea found a statistically significant association between ferritin and T2D risk [33], [34], [36]–[38], [41]. The analysis of the five case-control studies indicated that 2 studies showed a positive association between ferritin levels and T2D risk, though an additional study failed to confirm this result. The analysis of the remaining 2 case-control studies indicated a statistically significant association of the elevated transferrin saturation and the ratio of transferrin receptors to ferritin with T2D risk.

#### Findings of prospective studies

Six prospective studies (1 case-cohort, 3 nested case-control, 2 cohort), which analyzed the effect of ferritin levels on the incidence of T2D [10], [11], [13], [22], [25], [26], were included in the meta-analysis and had follow-up periods ranging from 2.8 to 10 years. The studies that were smaller than 300 T2D patients or were not adjusted for metabolic factors provided a statistically significant combined RR, while those that were larger than 300 or had adjusted for metabolic factors did not. Jiang *et al*. [10] and Forouhi *et al*. [26] evaluated large clinical populations (n = 698 and 360, respectively), reporting 2.6- and 3.2-fold increased risk for developing T2D compared to the groups with lowest ferritin levels after adjustment for confounders. Jehn *et al.* (n = 599) [13] initially reported a statistically significant association between ferritin levels and T2D risk (1.74, 95% CI: 1.14–2.65) when adjusted for age, study center, patient ethnicity, smoking and alcohol intake status of patients; however, they failed to find a statistically significant association (0.79, 95% CI: 0.48–1.32) after further adjustments for BMI and metabolic syndrome factors. Both Rajpathak *et al*. [22] and Salomaa *et al*. [11] found no statistically significant association between ferritin and T2D risk (1.61, 95% CI: 0.85–3.02, and 1.44, 95% CI: 0.93–2.24, respectively) after adjusting for multiple confounders, which was consistent with the findings of Le *et al*. [25] using female subjects (0.87, 95% CI: 0.37–2.03).

Two studies included in the qualitative analysis measured ferritin and transferrin saturation and found a statistically significant association of T2D risk with ferritin, but not with transferrin saturation, respectively. Thus, although there seems to be a consistent positive association between ferritin levels and T2D risk, we couldn’t completely rule out the possibility that ferritin may only serve as a mediator for metabolic abnormalities that directly contribute to T2D incidence. On the other hand, the meta-analysis of the association between heme-iron intake and T2D risk, which included four prospective cohort studies with larger than 1000 T2D cases with follow-up periods ranging from 8.8 to 20 years [12], [28]–[30], found a positive association between heme-iron intake and T2D risk. Considering that these four studies were well designed, the meta-analysis provided convincing combined results for the association of heme-iron intake and T2D incidence.

### Mechanisms

Genetic iron overload diseases, such as hereditary hemochromatosis, have been well recognized to contribute to diabetes by the excessive accumulation of iron in tissues [46]. Recent studies have mainly focused on the relationship between T2D and moderately elevated iron level that are lower than those in genetic iron overload diseases [47]. Researchers hypothesized that increased iron deposition in tissues initially induces insulin resistance, and the resulting hyperinsulinemia ultimately causes pancreatic islet apoptosis and the progression of T2D [48]. Through transforming poorly-reactive radicals into highly-active ones, catalytic iron damages DNA and the integrity of cell membrane, interferes with the glucose intake of skeletal muscles and adipocytes, and decreases the effect of insulin [6], [48], [49]. Indeed, ROS generated by catalytic iron inhibits insulin receptor function, resulting in impaired insulin uptake [50]. Increased iron stores further deteriorate hyperinsulinemia through hepatic dysfunction, decreased capacity for hepatic insulin extraction, and the insulin metabolism [5]. Ultimately, long-term over-secretion of insulin, iron deposition, and the hazardous effect of radicals on β-cells together contribute to β-cells apoptosis and T2D.

### Sources of Heterogeneity

Although the random-effect models were used to calculate the combined effect estimates, moderate and high heterogeneity was observed in prospective and cross-sectional studies regarding the association between ferritin levels and T2D risk, respectively. Thus, stratified analysis and meta-regression were introduced to explore the sources of heterogeneity.

#### Stratified analysis

Little or no heterogeneity was observed in prospective studies stratified by nested case-control (I^2^ = 2.6%, p = 0.358) or cohort studies (I^2^ = 0%, p = 0.559), and in the studies that had less than 300 T2D cases (I^2^ = 0%, p = 0.842), indicating that study design and size accounted for most of the heterogeneity among these prospective studies. The high heterogeneity of all combined cross-sectional studies was completely eliminated among the studies with less than 300 cases (I^2^ = 0%, p = 0.344), and partly attenuated among the studies that were adjusted for metabolic factors (I^2^ = 76.3%, p = 0.015) and the studies that used RIA to measure ferritin levels (I^2^ = 75.9%, p = 0.006).

#### Meta-regression

Considering the moderate heterogeneity in prospective studies, and high heterogeneity in cross-sectional studies, we further performed a meta-regression analysis to explore the potential sources of heterogeneity [51], [52]. Heterogeneity was completely eliminated by study design (single covariate: p = 0.026; residual I^2^ = 0%) in prospective studies, indicating that the three different study types could explain all the sources of heterogeneity. In cross-sectional studies, the country of origin where the studies were conducted had the potential to cause heterogeneity (single covariate: p = 0.125). The OR value of the only western study was 4.35 (95% CI: 3.00–6.31), which was significantly higher than the combined effect estimate of the other five Asian studies (1.98, 95% CI: 1.36–2.88). Other potential sources of heterogeneity could be study size (single covariate: p = 0.195; multiple covariates: p = 0.20) and metabolic factor adjustments (single covariate: p = 0.281). Meta-regression effectively explained the sources of heterogeneity in cross-sectional studies: without using meta-regression, the I^2^ value of heterogeneity was 88.7%; the residual I^2^ value was 49.94% when the study size and the adjustments were used as multiple covariates in the meta-regression; I^2^ value was further reduced to 33.3% when the number of patients and controls, and the study size were used as multiple covariates in the meta-regression.

### Limitations

Measurement errors, including the usage of different measurement methods for ferritin levels and varying times or measurement length may explain part of the variation among the prospective cohort study results. In addition, patients with T1D might have been included in the original studies as well; however, it is unlikely that the inclusion of T1D cases could influence the pooled RRs significantly, considering that the prevalence of T1D is less than 5–10% in adult diabetes patients. Languages might explain part of the heterogeneity, because all the included articles were published in English. However, articles in other languages were also read, if their abstracts were provided in English, which reduced the likelihood of incomplete retrieval of results. Furthermore, publication bias might also influence the pooled RRs, but our Begg and Egger tests did not indicate any potential publication bias.

The results of our meta-analysis were unlikely to be explained by residual confounding due to the strength of the adjusted RRs for ferritin levels and the risk of T2D. The higher combined RR for ferritin levels in cross-sectional studies compared with prospective studies suggests the likelihood of reverse causality. However, the difference in pooled RR between the two study designs was not statistically significant and the strength of RR for prospective studies was high, indicating that reverse causality was unlikely. Since all the included studies were large population-based studies and 4 out of 5 were prospective cohort studies, residual confounding could be ruled out regarding our results for the association of T2D risk with heme-iron intake. Larger prospective studies including both genders, patients from diverse geographic areas, and adjustments for metabolic factors are needed in the future to establish a more definitive conclusion about the association of T2D risk with ferritin levels and heme-iron intake.

### Validity of the Meta-analysis

The studies included in the meta-analysis, assessing the association between ferritin levels and T2D risk, were high- (prospective studies) and moderate-quality (cross-sectional studies). The high to moderate quality of all included studies for quantitative analysis ensured the validity of the meta-analysis. High heterogeneity existed among the cross-sectional studies, indicating actual variation rather than pure chance in study results, which was expected, given the diverse ethnic background of patients, inconsistent criteria used for T2D diagnosis, and the varied ferritin measurement methods. Although the results of the stratified analysis and meta-regression indicated that residual confounding among cross-sectional studies might exist, nested case-control and cohort studies were homogeneous. Besides, to exclude the likelihood that ferritin merely served as a surrogate marker for the association with T2D, we included a wide range of metabolic factors, such as HDL-C, triglycerides, CRP, adiponectin, adipokines, liver enzymes, inflammation score, insulin levels, and dietary factors, in our meta-analysis. We calculated the combined RRs of prospective studies before and after the adjustments of metabolic factors. Under both conditions, prospective studies indicated a statistically significant association between ferritin levels and T2D risk, suggesting a causal effect for high ferritin level on T2D independent of known diabetes risk factors.

### Conclusion

In summary, our systemic review and meta-analysis suggest that higher ferritin levels and heme-iron intake were both associated with an increased risk of T2D. Additional large prospective studies adjusted for metabolic factors are needed to confirm this causal relationship.

## Supporting Information

Table S1 **PRISMA Checklist.**
(DOC)
